# Ultrasound of acquired posterior fossa abnormalities in the newborn

**DOI:** 10.1038/s41390-020-0778-9

**Published:** 2020-03-26

**Authors:** Monica Fumagalli, Alessandro Parodi, Luca Ramenghi, Catherine Limperopoulos, Sylke Steggerda, eurUS.brain group, Thais Agut, Ana Alarcon, Roberta Arena, Marco Bartocci, Mayka Bravo, Fernando Cabañas, Nuria Carreras, Olivier Claris, Dudink, Monica Fumagalli, Paul Govaert, Sandra Horsch, Alessandro Parodi, Adelina Pellicer, Luca Ramenghi, Charles C. Roehr, Sylke Steggerda, Eva Valverde

**Affiliations:** 1 NICU, Fondazione IRCCS Ca‘ Granda Ospedale Maggiore Policlinico, Milan, Italy; 2 University of Milan, Department of Clinical Sciences and Community Health, Milan, Italy; 3 Neonatal Intensive Care Unit, Istituto Giannina Gaslini, Via Gaslini 5, 16148 Genoa, Italy; 4 Developing Brain Research Laboratory, Departments of Diagnostic Imaging and Radiology, Children’s National Health System, Washington, DC USA; 5 Department of Neonatology, Leiden University Medical Center, Leiden, The Netherlands; 6 Department of Neonatology, Hospital Sant Joan de Déu, Institut de Recerca Sant Joan de Déu, Barcelona, Spain; 7 Catholic University of the Sacred Heart, A. Gemelli Hospital, Rome, Italy; 8 Department of Women’s and Children’s Health, Karolinska University Hospital, Karolinska Insitute, Stockholm, Sweden; 9 Department of Neonatology, La Paz University Hospital, Madrid, Spain; 10 Department of Neonatology, Quironsalud Madrid University Hospital and Biomedical Research Foundation, La Paz University Hospital, Madrid, Spain; 11 Service de néonatologie et de réanimation néonatale, Hospices Civils de Lyon, Université Claude Bernard Lyon, Villeurbanne, France; 12 UMCU-Wilhelmina Children’s Hospital, Lundlaan 6, 3584 EA Utrecht, The Netherlands; 13 Department of Neonatology, Sophia Children’s Hospital, Erasmus Medical Center University, Rotterdam, The Netherlands; 14 Department of Neonatology, ZNA Middelheim, Antwerp, Belgium; 15 Department of Rehabilitation and Physical Therapy, Gent University Hospital, Gent, Belgium; 16 Department of Neonatology, Helios Klinikum Berlin Buch, Berlin, Germany; 17 Department Clinical Science Intervention and Technology (CLINTEC), Karolinska Institutet, Stockholm, Sweden; 18 Department of Paediatrics, Medical Sciences Division, Newborn Services, University of Oxford, Oxford, UK

## Abstract

Neonatal brain sonography is part of routine clinical practice in neonatal intensive care units, but ultrasound imaging of the posterior fossa has gained increasing attention since the burden of perinatal acquired posterior fossa abnormalities and their impact on motor and cognitive neurodevelopmental outcome have been recognized. Although magnetic resonance imaging (MRI) is often superior, posterior fossa abnormalities can be suspected or detected by optimized cranial ultrasound (CUS) scans, which allow an early and bed-side diagnosis and monitoring through sequential scans over a long period of time. Different ultrasound appearances and injury patterns of posterior fossa abnormalities are described according to gestational age at birth and characteristics of the pathogenetic insult. The aim of this review article is to describe options to improve posterior fossa sequential CUS image quality, including the use of supplemental acoustic windows, to show standard views and normal ultrasound anatomy of the posterior fossa, and to describe the ultrasound characteristics of acquired posterior fossa lesions in preterm and term infants with effect on long-term outcome. The limitations and pitfalls of CUS and the role of MRI are discussed.

## Introduction

Imaging the neonatal brain is part of clinical routine practice in neonatal medicine, and sequential cranial ultrasound (CUS) scans allow detection of most of the typical lesions, which correlate with adverse prognosis.^1,2^ The neuroimaging study of the posterior fossa is gaining increasing attention due to the relative incidence of posterior fossa abnormalities (mainly in preterm infants) and to the broad spectrum of associated neurodevelopmental impairments involving cerebral “higher-order functions” such as memory, language, reading ability, and cognitive performances.^3,4^ The rapidly developing cerebellum of the preterm infant is a very vulnerable brain structure and both cerebellar hemorrhage (CBH) and impaired cerebellar development can therefore complicate preterm birth.^5^ In the (near) term infant cerebellar injury and extra axial posterior fossa hemorrhage can also occur, that is, in cases with traumatic delivery, infection, inflammation, and perinatal asphyxia.^6,7^ However, on routine CUS images obtained through the anterior fontanel (AF) visualization of the posterior fossa (and in particular of the cerebellum) is often poor.^8,9^

Clinicians have used CUS to detect brain injury in newborn infants since the 1970s, but only in the past decade CUS of the posterior fossa has gained increasing attention. The aim of this review article is to discuss the applications and indications of CUS of the neonatal posterior fossa; this review is based on personal experience and a selection of papers on posterior fossa ultrasound available in PubMed, most of which have been used as references. We (i) describe options to improve CUS image quality, including the use of supplemental acoustic windows; (ii) show illustrations of the normal ultrasound anatomy of the posterior fossa; (iii) describe the ultrasound characteristics of acquired posterior fossa lesions in the newborn; (iv) discuss the limitations and pitfalls of CUS and the role of magnetic resonance imaging (MRI).

## Ultrasound of the neonatal posterior fossa: technical aspects and normal anatomy

In standard neonatal CUS examination the AF is used as acoustic window. This approach provides good visualization of the supratentorial region located close to the transducer (see related chapter on technique in this seminar). When scanning through the AF, the area most poorly visualized is the posterior fossa. This is mainly because the posterior fossa is located furthest away from the transducer. In addition, the insonation angle and the echogenic tentorium can impede image quality. However, several types of lesions can still be suspected by insonation through the AF. The options to improve CUS of the neonatal posterior fossa are discussed below.

### Adaptation of settings

**Fig. 1:**
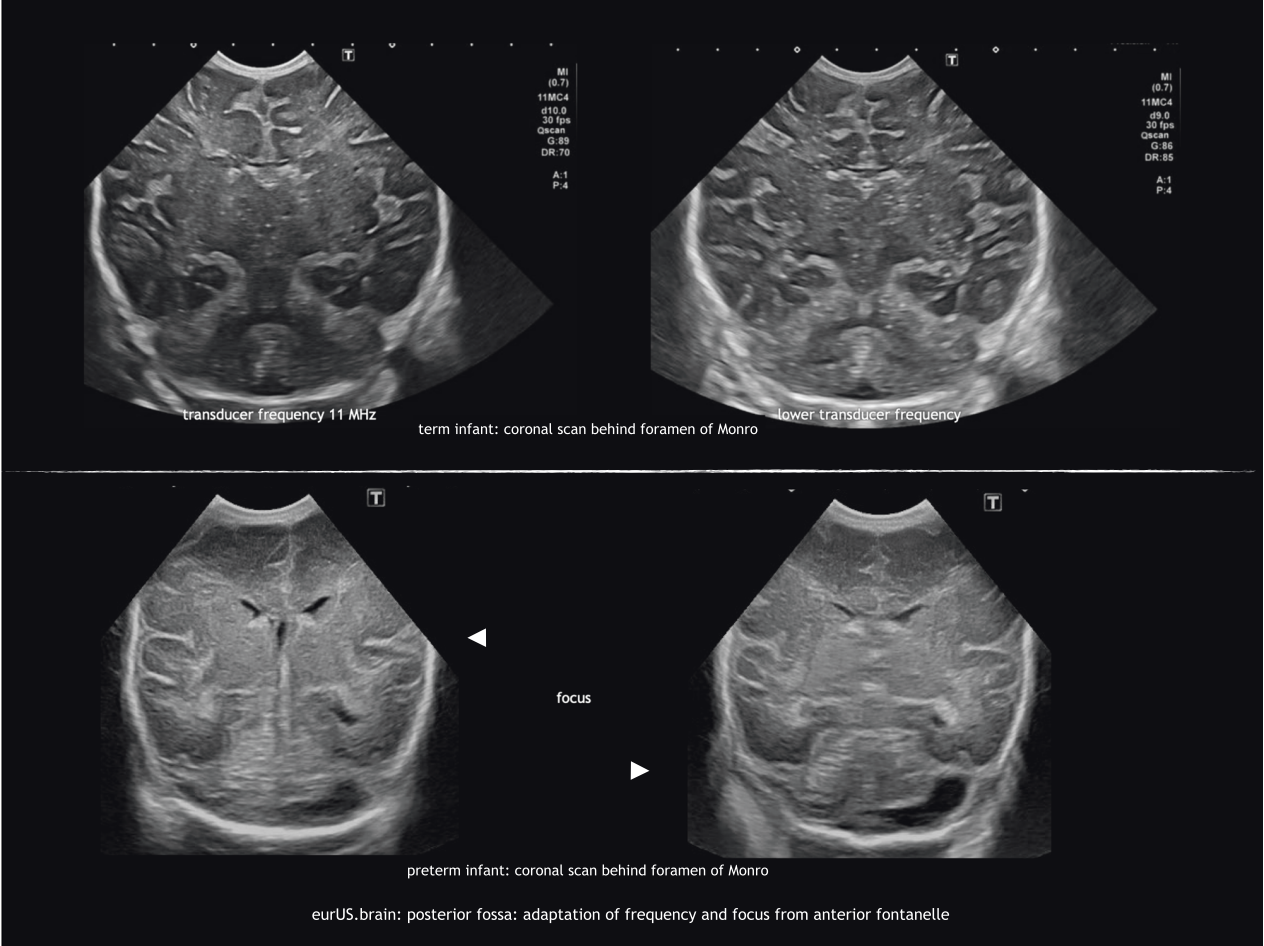
Posterior fossa: adaptation of frequency and focus from anterior fontanel.

The standard transducer frequency for neonatal CUS is 8–11 MHz. This frequency provides high-resolution images of the ventricular system, periventricular white matter, and deep gray matter structures. In extremely small, preterm infants, these settings also allow visualization of the posterior fossa, but in larger infants penetration is often insufficient. As a first step to improve image quality, one or more focus points can be aimed at the infratentorial region. In addition, decreasing the transducer frequency (5–6 MHz) will result in a better penetration of the ultrasound beam. However, lower frequencies reduce the resolution of the ultrasound image (Fig. 1).^10^

### The use of additional acoustic windows

#### The mastoid fontanel

Several articles have recommended imaging through the mastoid fontanel (MF), also referred to as posterolateral fontanel, to improve ultrasound imaging of the neonatal posterior fossa.^9–11^ With this approach the transducer is placed closer to the posterior fossa structures. Therefore, higher transducer frequencies can be used (8–11 MHz with the standard microconvex probes and even higher, up to 15–18 MHz, with linear probes), thus improving the resolution of the images. Furthermore, the structures are approached from underneath the tentorium and at a more perpendicular angle instead of parallel to the ultrasound beam. This leads to a better visualization of the posterior fossa structures and to a better detection of abnormalities.^12–14^ The MFs are located at the junction of the parietal, temporal, and occipital bones^8–11,15^ When performing the examination, the sonographist places the transducer in the mastoid area, behind the helix of the ear and then slightly moves and rotates it to obtain a clear and symmetric view of the posterior fossa. Images are recorded in axial (also transverse) and coronal planes, at different levels.^9,10^ Cerebellar hemispheres, cerebellar vermis, cisterna magna, the fourth ventricle, and its plexus can be easily visualized through the MF.^9,10,16^ In addition, the transcerebellar diameter can be measured. The use of color Doppler allows visualization of venous flow in the transverse and sigmoid sinuses: this can be useful in the diagnosis of sinovenous thrombosis.^17,18^ In our experience, neonates can show some signs of discomfort during MF sonography. This may be explained by an auditory response to pulses of radiofrequency energy.^19^ Therefore, we advise to perform these views at the end of the CUS examination. Often the whole posterior fossa can be scanned through one single side MF (the one most easily accessible). The opposite MF can be used to confirm or exclude any suspected abnormalities, during the same or a subsequent examination. Usually the approach only costs a few minutes of additional scanning time.

#### The posterior fontanel

**Fig. 2:**
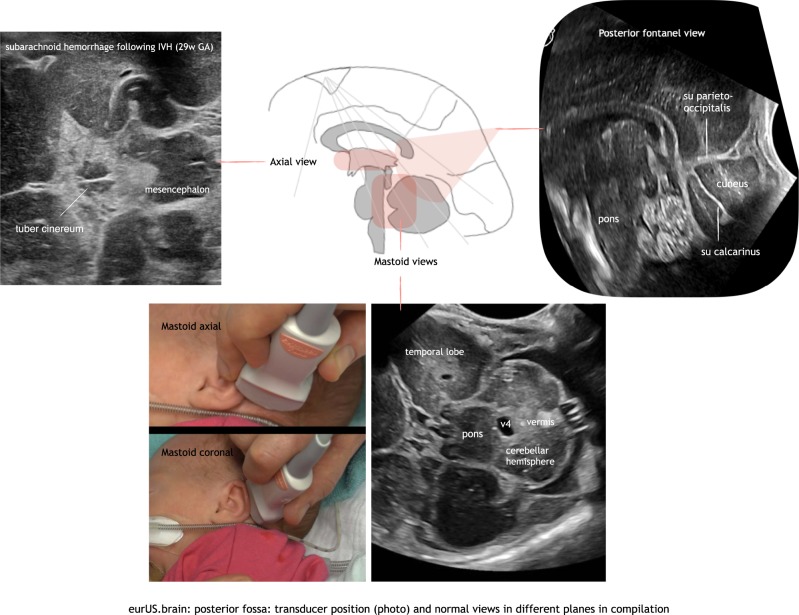
Posterior fossa: transducer position (photo) and normal views in different planes in compilation.

**Fig. 3:**
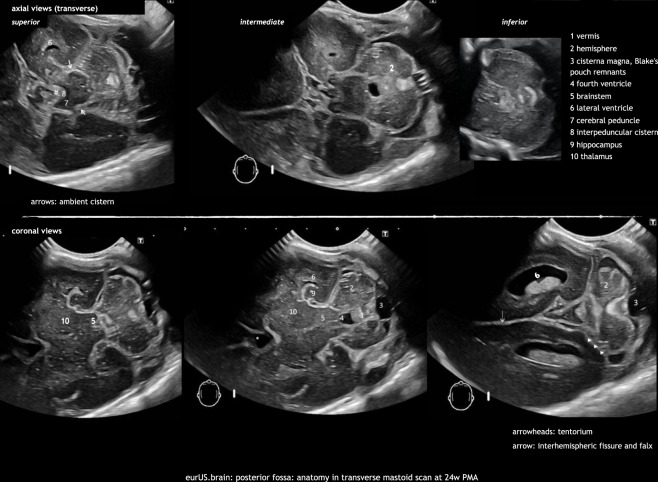
Posterior fossa: anatomy in transverse mastoid scan at 24 weeks PMA.

The posterior fontanel (PF) is located at the junction of the lambdoid and sagittal sutures.^11,15^ PF CUS is especially useful in the diagnosis of low-grade germinal matrix-intraventricular hemorrhage (GMH-IVH) and lesions in the occipital parenchyma, and also helps to define posterior fossa anatomy.^20^ PF CUS includes both coronal and sagittal views. Especially the midsagittal section provides a more detailed view of the cerebellar vermis, fourth ventricle, and cisterna magna (Figs. 2 and 3).

## Acquired lesions of posterior fossa

### Cerebellar hemorrhage in preterm infants

CBH is the most frequently observed acquired injury of the posterior fossa in preterm infants, with the most immature infants bearing the highest risk. It is supposed to originate from the external granular layer covering the cerebellar surface as well as from the germinal matrix located in the subependymal layer of the roof of the fourth ventricle,^21^ although recent autopsy studies support a possible origin from the densely vascularized region of the emerging internal granule cell layer and adjacent white matter.^22,23^ The pathogenesis of CBH is known to be multifactorial and several risk factors have been identified: emergency cesarean section, patent ductus arteriosus, and lower 5-day minimum pH.^24^ Different CBH patterns have been described in preterm infants by neuropathology and neuroimaging studies:

i. Microhemorrhages within the hemispheric parenchyma. These punctate hemorrhagic lesions are usually undetectable by CUS (even when scans are performed through the MF), but they are easily detected by MRI, especially when the susceptibility weighted imaging (SWI) sequence is used.^12,13^ However, the prognosis of these punctate lesions is still debated.^25,26^
ii. Large CBH is primarily unilateral and located in the parenchyma of the cerebellar hemispheres; the vermis is involved in <1/3 of cases^24^ mainly when bleeding occurs within the germinal matrix located in the subependymal layer of the roof of the fourth ventricle. Large CBHs may be focal and of limited size (usually >4 mm but involving less than 1/3 of the cerebellar parenchyma). They can be detected on CUS, especially when MF views are performed. Often they are located at the lateral convexity of the cerebellar hemispheres, and on follow-up ultrasound or MRI there may be slight atrophy or irregularity of the affected hemisphere. Extensive hemorrhages involve more than 1/3 of the cerebellar hemisphere and/or vermis and often lead to clear atrophy on follow-up scans. They can be diagnosed on MF views, but are often also visible on AF views when attention is paid to the posterior fossa. Extensive CBHs are more likely to occur in the youngest and sickest infants and are associated with the worst prognosis.^27,28^

**Fig. 4:**
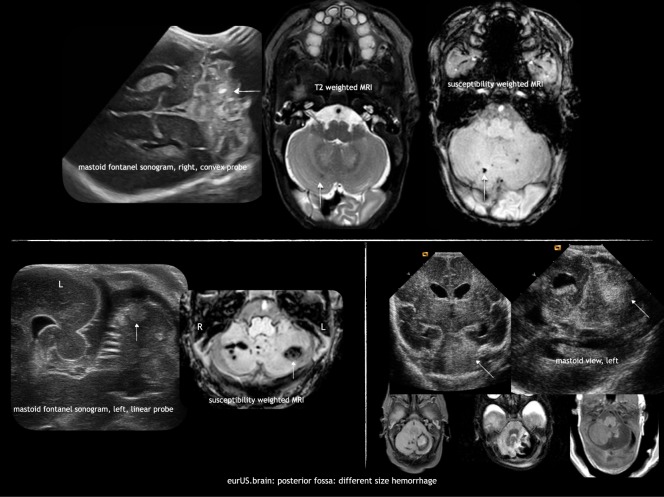
Posterior fossa: different size hemorrhage.

Different ultrasound features of large focal CBHs can be observed according to timing. Acute phase: a globular or less circumscript area of increased echogenicity is spotted within the cerebellar parenchyma, with possible concurrent ventricular dilatation, even in the absence of GMH-/IVH; subacute phase: less echogenic and even echolucent lesions are observed; chronic phase: focal or extensive atrophy of the cerebellum is measured. A flattening of the pontine base can be also appreciated on late scans. A frequent association with supratentorial GMH-IVH has been reported; therefore, the occurrence of CBH in infants with GMH-IVH needs to be carefully investigated. Parodi et al.^13^ found that 67.8% of all CBHs were associated with GMH-IVH; similar results were reported by Steggerda et al.^12^. Among patients with microhemorrhages, intraventricular hemosiderin deposits related to GMH-IVH were observed in 65.0% of infants^13^ and the authors also hypothesized that SWI detected hypointensities on cerebellar surface might represent, in some cases, hemosiderin depositions originating from supratentorial bleeding (Fig. 4).

### Impaired cerebellar development in very preterm infants

Preterm birth has been associated with impaired cerebellar growth even in the absence of obvious cerebellar lesions.^5^ The disturbance in development is inversely related to gestational age at birth^29^ and again different mechanisms are involved: hypoxia–ischemia, infection–inflammation, poor nutrition, and glucocorticoid exposure, as well as indirect remote effects related to hemosiderin deposits originating from IVH.^5,30–32^ At the MF transverse cerebellar diameter can be measured in both axial and coronal plane, to be compared with nomograms available for different gestational ages for detecting cerebellar hypoplasia or atrophy.^33–35^

### Cerebellar hemorrhage in the term infant

**Fig. 5:**
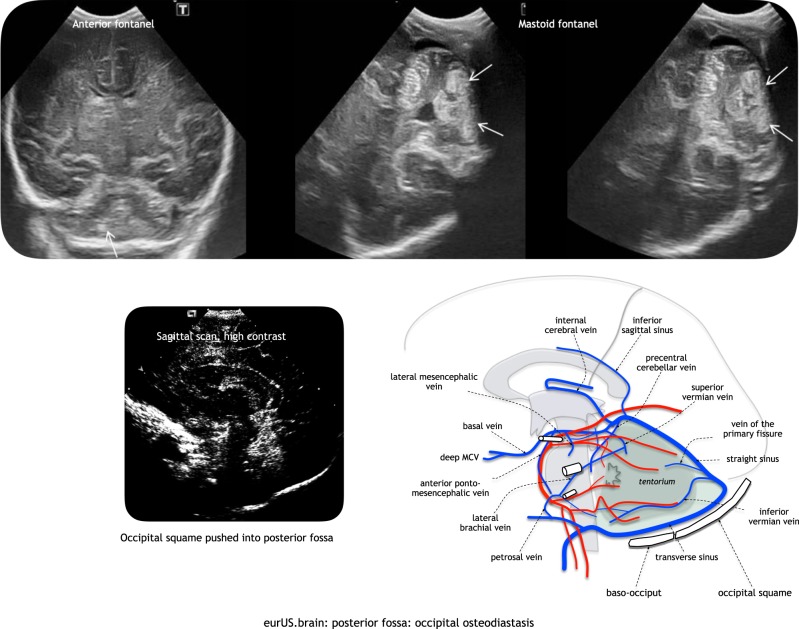
Posterior fossa: occipital osteodiastasis. Arrows indicate CBH. Relation of bone to posterior fossa veins.

Cerebellar hemorrhagic lesions are less common but may also occur in (near) term infants and include both small or punctate hemorrhages deep within the cerebellum and large hemispheric or vermian hemorrhage.^6^ Prenatal and intrapartum risk factors observed in term infants with cerebellar injury include primiparity, advanced maternal age, group B S*treptococcus*-positive mothers, abnormal fetal heart rate, instrumented delivery, and cesarean section. They may be caused by occipital osteodiastasis related to traumatic delivery and/or breech presentation:^36^ in this case, hemorrhagic lesions mainly involve the inferior cerebellar areas and have been described as contusions (Fig. 5). Hemorrhagic lesions may also occur as a consequence of underlying systemic hemorrhagic disorders, due to heparinization required for invasive procedures (like extracorporeal membrane oxygenation) and/or due to increases in cerebral venous pressure. Cerebellar injury in term infants is associated with a broad spectrum of neurodevelopmental disabilities, including gross motor delay, language deficits, and behavioral problems.^6^ The size of the lesion(s), presence of cerebellar hemispheric atrophy, and vermis involvement are associated with the degree of impairment.

### Fetal cerebellar hemorrhage

**Fig. 6:**
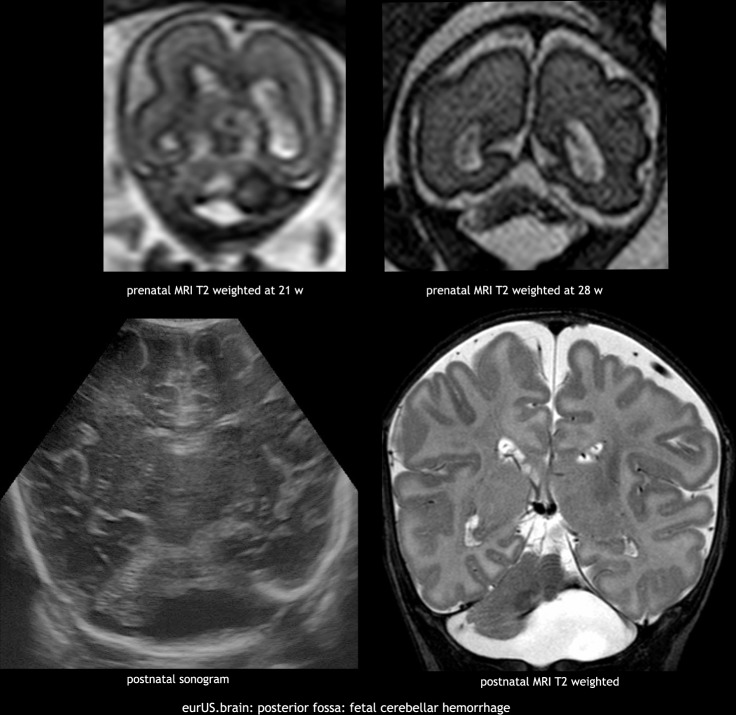
Posterior fossa: fetal cerebellar hemorrhage.

Isolated fetal CBH is a moderately rare event occuring early in fetal life. Fetal MRI studies suggest a prevalent origin in lateral and caudal portions of the cerebellar hemispheres,^37^ although the pathogenesis is still debated: fetal distress, growth restriction, placental thrombotic vasculopathy, fetal infection (parvovirus^38^ or cytomegalovirus [CMV]^39^), anemia with/without intrauterine transfusion,^40^ twin to twin transfusion syndrome, hydrops, and maternal conditions (thrombophilia, septic shock^41,42^) have been reported as associated factors. Martino et al.^37^ suggested a venous origin, similar to supratentorial GMH-IVH, associated with deep venous system engorgement due to different systemic conditions. On postnatal CUS, fetal CBH lesions usually appear at a chronic stage characterized by focal hemispheric volume reduction and distortion and they may also mimic cerebellar malformation. To date, information of neurodevelopment after disruptive fetal cerebellar hemorrhage is lacking^43^ due to the high rate of termination of pregnancy. Aziz et al.^40^ reported a favorable short-term prognosis, but a high incidence of language disorders has been observed^37^ as well as cognitive and behavioral impairment^42^ (Fig. 6).

## Extra-cerebellar posterior fossa hemorrhage

**Fig. 7:**
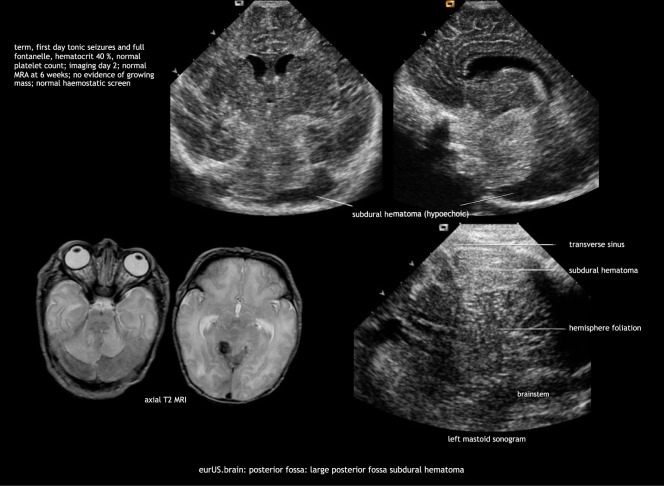
Posterior fossa: large posterior fossa subdural hematoma.

Minor subdural hemorrhages are commonly detected by MRI in asymptomatic term infants after vaginal delivery, usually they are undetectable by CUS.^44^ Major subdural hemorrhage in the newborn can occur above and below the tentorium and is often the result of traumatic delivery. Major infratentorial hemorrhage occurs after tentorial laceration—with rupture of the straight sinus, vein of Galen, transverse sinus, or infratentorial veins—and after occipital osteodiastasis with rupture of the occipital or other sinus. Affected infants usually develop neurologic symptoms related to brainstem or cranial nerve compression. Early recognition is crucial because deterioration can be rapid and prompt surgical intervention can be lifesaving. In general, computed tomography (CT) or MRI are considered superior to CUS to demonstrate the extent of the lesions. However, there are clear ultrasound features of major posterior fossa hemorrhage both on AF and MF views. Recognition of these features facilitates early diagnosis and timely intervention. Features of major posterior fossa hemorrhage on AF views are: unexplained ventricular dilatation; apparent broadening of the “tentorium”; difficulty to delineate midline posterior fossa structures (cerebellar vermis, pons) or an area of increased echogenicity above the vermis in midsagittal plane. Features on MF views are: mass effect on cerebellum with shift of one or both hemispheres, compression of the fourth ventricle; extra-axial blood surrounding cerebellar hemisphere(s) and/or between hemisphere and tentorium (of note: acute hemorrhage often has an echolucent appearance); thickening of the perimesencephalic cisterns indicating subarachnoid bleeding (Fig. 7).

## Ventricular dilatation

**Fig. 8:**
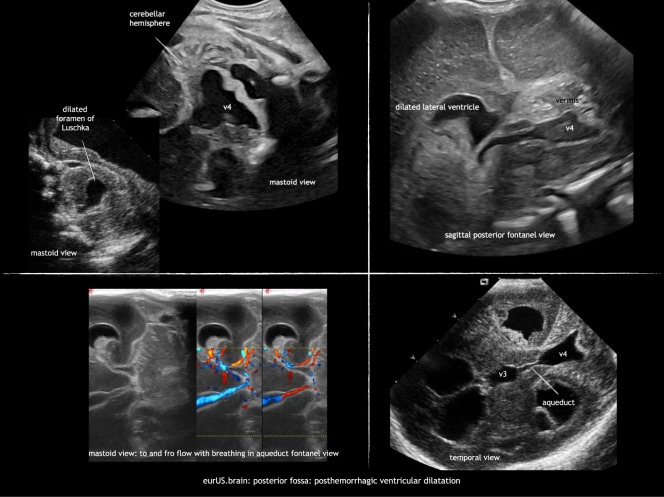
Posterior fossa: posthemorrhagic ventricular dilatation.

Ventricular dilatation can occur as a sequela of GMH-IVH or intracranial infection. It is due to obstruction by a clot or subependymal scarring at the level of the foramen of Monro, aqueduct, fourth ventricle exit foramina, or cerebellar arachnoid spaces. Scanning through the MF visualizes the ventricular system and locates the site of obstruction. A dilated aqueduct—in some with to and fro motion on Doppler exam—and/or fourth ventricle can be visualized by ultrasound (Fig. 8). When both the inflow and outflow of the fourth ventricle are obstructed, this can lead to a severely dilated trapped fourth ventricle, with brainstem compression. This requires a specific therapeutic approach.

## Miscellaneous

In the sick newborn infant the cerebellum can also be involved in other situations, such as hypoxic–ischemic conditions, central nervous system infection, and metabolic disorder. This affects both preterm and term infants. The cerebellar abnormalities are often diagnosed by MRI. However, CUS is useful (especially when scans are performed using the MF) as  it enables early diagnosis of large, clinically relevant lesions.^14^

### Hypoxic–ischemic injury

The cerebellum is vulnerable to hypoxic–ischemic injury. The most vulnerable neurons are the Purkinje cells (in term infants) and the granule cell neurons of both the internal and external granular layers (in preterm and full-term infants). In preterm infants, hypoxic–ischemic conditions have been related to bilateral and symmetrical cerebellar injury of especially the hemispheres, which can result in smaller cerebellar volumes in later life.^5,45^ In term infants the vermis is susceptible to injury and a subsequent disturbance of vermis growth has been reported on follow-up MRI scans.^46–48^ Because these lesions often occur in combination with injury to basal ganglia and thalamus, it is not clear whether subsequent growth failure is due to direct effects or occurs secondary to supratentorial brain damage. However, cerebellar abnormalities have also been reported on early neonatal MRI scans and in combined MRI/postmortem studies in infants with severe hypoxic–ischemic encephalopathy.^49–51^ On CUS the acute phase of hypoxic–ischemic cerebellar injury is difficult to visualize, but with extensive damage increased echogenicity of the cerebellar hemispheres and/or vermis and a loss of foliation can be seen using the MF. Rarely cavitation in the core of cerebellar white matter is observed.

### Infection

Congenital CMV infection can result in disturbances of neuronal proliferation and migration, thus cerebellar hypoplasia is common in symptomatic cases. Apart from characteristic ultrasound abnormalities, such as subependymal germinal matrix cysts, calcification, and striatal arteriopathy, cerebellar hypoplasia may be an additional CUS finding suggestive of congenital CMV.^45,52^

**Fig. 9:**
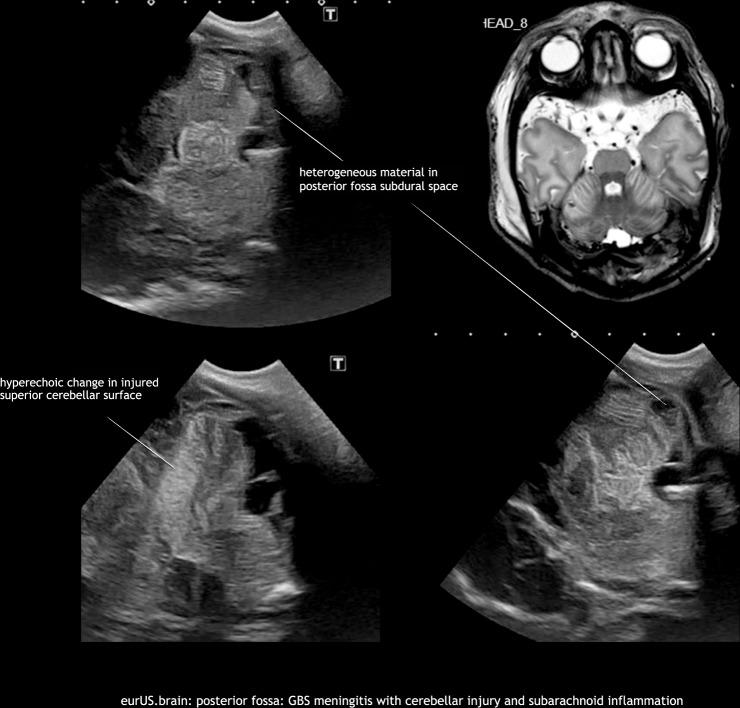
Posterior fossa: Group B *Streptococcus* meningitis with cerebellar injury and subarachnoid inflammation.

**Fig. 10:**
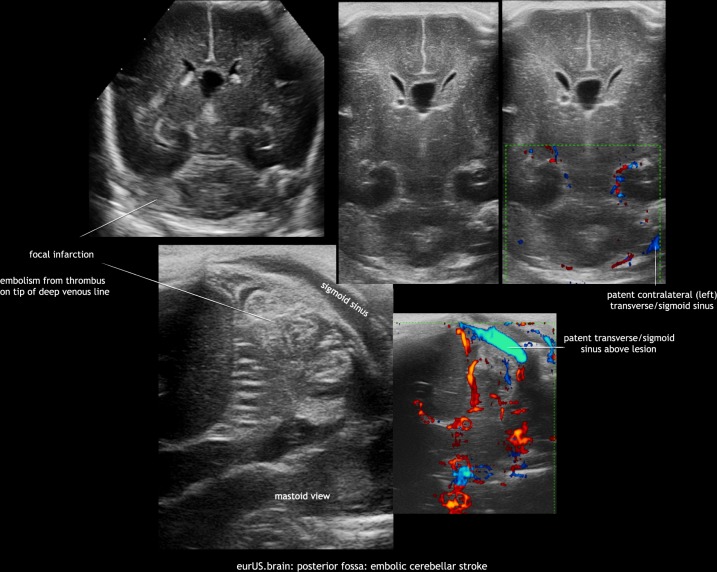
Posterior fossa: embolic cerebellar stroke.

Neonatal bacterial meningitis may be complicated by arterial or venous cerebral infarction. Lesions are often located supratentorially, but have also been described within the cerebellum, especially in cases with group B *Streptococcus* meningitis. In the acute phase this may appear as increased echogenicity of the involved cerebellar structures on ultrasound. It may progress to destruction and atrophy on follow-up imaging.^7^ Bacterial meningitis can be complicated by an impairment of cerebrospinal fluid flow and hydrocephalus. In some cases, this occurs in combination with a dilated or isolated fourth ventricle. Brain abscess is an uncommon but very serious complication of newborn central nervous system infections; most are located supratentorially. However, a few cases with cerebellar abscesses have been reported, often in preterm infants with, that is, *Staphylococcus aureus* or disseminated fungal infections. Most were studied by CT or MRI, but CUS may also be a valuable tool to detect these lesions.^9,20^ Their sonographic appearance varies depending on organism and evolution with time. Typical findings include single or multiple round hypoechoic lesions surrounded by hyperechoic borders. Embolic focal infarcts may or may not be infected (Figs. 9 and 10).

### Inborn errors of metabolism

Several inborn errors of metabolism can affect the developing cerebellum and lead to structural abnormalities and hypoplasia. Depending on timing of the insult, they may present with progressive cerebellar hypoplasia during pregnancy or in early postnatal life. Examples of metabolic disorders presenting with fetal or early neonatal cerebellar atrophy are carbohydrate-deficient glycoprotein syndrome, Zellweger syndrome, adenylsuccinate lyase deficiency, pyruvate dehydrogenase complex deficiency, and other mitochondrial disorders.^53,54^

## Limitations and pitfalls of posterior fossa ultrasound and the role of MRI

### Limitations of posterior fossa ultrasound

Imaging the preterm brain through the AF is part of clinical routine practice. Adding MF views to standard CUS protocols increases detection of posterior fossa abnormalities and enables early diagnosis of large and clinically relevant lesions. It offers advantages compared to other imaging techniques but it also has limitations: it is operator dependent; the quality of imaging is affected by the anatomical characteristics of the acoustic windows (small windows and Continuous Positive Airway Pressure hats may result in low quality); in unexperienced hands adding MF views may prolong scanning time and increase discomfort to the infants.

### Pitfalls of posterior fossa ultrasound

When performing CUS through the MF one should be aware of some pitfalls. A false diagnosis of inferior vermis agenesis can be suspected based on the appearance of a normal but large communication between the fourth ventricle and the cisterna magna in the most posterior coronal plane: this suspicion needs to be confirmed by a mid-sagittal scan through the AF. When scanning the cerebellum through one MF, an area of apparent increased echogenicity may be observed in the controlateral cerebellar hemisphere: increased echogenicity due to bone structures has to be excluded by scanning the posterior fossa from the opposite MF before rising the suspicion of cerebellar hemorrhage. Several days after an acute hemorrhagic event, limited CBH may become undetectable by CUS: early and sequential CUS scans are important not to miss CBH diagnosis. At higher gestational ages visualization of both cerebellar hemispheres from one-side MF may become challenging (due to increased cerebellar size); scanning through both MFs is advisable in these cases to avoid false diagnosis of hemispheric cerebellar hypoplasia.

### The role of MRI when imaging the posterior fossa

**Fig. 11:**
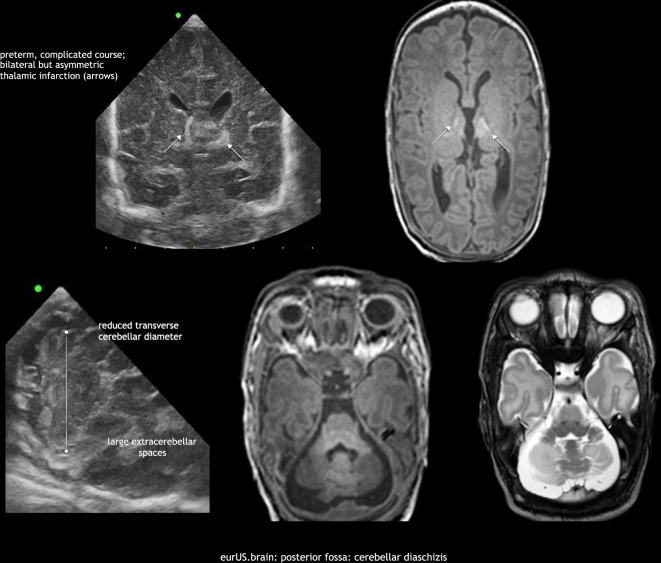
Posterior fossa: cerebellar diaschisis.

MRI is superior to CUS in identifying and defining the extension of brain abnormalities occurring in the posterior fossa, in particular when CUS is limited to AF scanning.^12,13^ MF scanning enables early detection of abnormal findings in the posterior fossa in high-risk term infants and has to be performed although its sensitivity is about 57% and specificity 95% compared to MRI.^14^ Small punctate CBHs (<4 mm) are reported as a common finding at MRI in very preterm infants (especially when SWI is used),^13^ while they usually remain undetected by CUS in spite of the routine use of MF.^12^ Although the clinical relevance of these small-sized CBHs is controversial,^25,26^ this finding is reported in about 8–20% of preterm infants <34 weeks gestation.^12,13,25,26,55^ MRI also allows better detection of associated supratentorial abnormalities, such as low-grade GMH-IVH that can be underdiagnosed by CUS.^56^ Interestingly, advanced quantitative MRI techniques have well-documented detrimental effects of preterm birth on global and regional brain growth, even in the absence of direct cerebral/cerebellar parenchymal injury, by showing impaired cerebellar development in preterm newborns at term compared with in utero healthy fetuses.^57^ The cerebellum is indirectly richly connected with regions of the contralateral cerebral cortex. Advanced MRI techniques have brought increasing evidence that early-life cerebellar injury influences the development of structure and function of specific cerebral cortical areas, for example, involved in motor function, cognition, language, and behavior. MRI can provide further insight into these structure–function relationships and into the mechanisms by which early cerebellar injury contributes to neurodevelopmental impairment (diaschisis)^58–60^ (Fig. 11). Nonetheless, the use of such advanced techniques in a routine clinical setting is still not feasible.

## Conclusion

CUS represents a valuable tool to investigate the posterior fossa in newborn infants. Optimal settings, probe choice, and specific acoustic windows influence its sensitivity in diagnosing and interpreting lesions. MRI allows a better identification of abnormalities and definition of their extension; advanced MRI definitely contributes to shed light on the pathogenesis and long-term correlates of early-life cerebellar injury.

## References

[1] Brouwer MJ (2014). Sequential cranial ultrasound and cerebellar diffusion weighted imaging contribute to the early prognosis of neurodevelopmental outcome in preterm infants. PLoS ONE.

[2] de Vries LS, Benders MJNL, Groenendaal F (2013). Imaging the premature brain: ultrasound or MRI?. Neuroradiology.

[3] Brossard-Racine M, du Plessis AJ, Limperopoulos C (2015). Developmental cerebellar cognitive affective syndrome in ex-preterm survivors following cerebellar injury. Cerebellum.

[4] Limperopoulos C (2007). Does cerebellar injury in premature infants contribute to the high prevalence of long-term cognitive, learning, and behavioral disability in survivors?. Pediatrics.

[5] Volpe JJ (2009). Cerebellum of the premature infant: rapidly developing, vulnerable, clinically important. J. Child Neurol..

[6] Limperopoulos C, Robertson RL, Sullivan NR, Bassan H, du Plessis AJ (2009). Cerebellar injury in term infants: clinical characteristics, magnetic resonance imaging findings, and outcome. Pediatr. Neurol..

[7] Steggerda SJ, de Bruïne FT, Smits-Wintjens VEHJ, Walther FJ, van Wezel-Meijler G (2012). Ultrasound detection of posterior fossa abnormalities in full-term neonates. Early Hum. Dev..

[8] Buckley KM (1997). Use of the mastoid fontanelle for improved sonographic visualization of the neonatal midbrain and posterior fossa. Am. J. Roentgenol..

[9] Enriquez G (2006). Mastoid fontanelle approach for sonographic imaging of the neonatal brain. Pediatr. Radiol..

[10] Steggerda SJ, van Wezel-Meijler G (2016). Cranial ultrasonography of the immature cerebellum: role and limitations. Semin. Fetal Neonatal Med..

[11] Di Salvo DN (2001). A new view of the neonatal brain: clinical utility of supplemental neurologic US imaging windows. Radiographics.

[12] Steggerda SJ (2009). Cerebellar injury in preterm infants: incidence and findings on US and MR images. Radiology.

[13] Parodi A (2015). Accuracy of ultrasound in assessing cerebellar haemorrhages in very low birthweight babies. Arch. Dis. Child.

[14] Steggerda SJ (2015). Posterior fossa abnormalities in high-risk term infants: comparison of ultrasound and MRI. Eur. Radiol..

[15] Meijler, G. Steggerda, S.J. *Neonatal Cranial Ultrasonography, 3rd edition* (Springer, Nature, Switzerland, 2019).

[16] Teele RL, Taylor GA (2012). Demonstration of fourth ventricular choroid plexus on neonatal cranial ultrasonography. Pediatr. Radiol..

[17] Raets MMA (2013). Serial cranial US for detection of cerebral sinovenous thrombosis in preterm infants. Radiology.

[18] Miller E (2012). Color Doppler US of normal cerebral venous sinuses in neonates: a comparison with MR venography. Pediatr. Radiol..

[19] Elder, J. A. & Chou, C. K. Auditory response to pulsed radiofrequency energy. *Bioelectromagnetics*10.1002/bem.10163 (Suppl. 6), S162–S173 (2003). 10.1002/bem.10163 14628312

[20] Correa F (2004). Posterior fontanelle sonography: an acoustic window into the neonatal brain. Am. J. Neuroradiol..

[21] Biran, V., Verney, C. & Ferriero, D. M. Perinatal cerebellar injury in human and animal models. *Neurol. Res. Int.* (2012). http://www.hindawi.com/journals/nri/2012/858929. 10.1155/2012/858929 PMC3317029 22530126

[22] Pierson CR, Al Sufiani F (2016). Preterm birth and cerebellar neuropathology. Semin. Fetal Neonatal Med..

[23] Haines KM, Wang W, Pierson CR (2013). Cerebellar hemorrhagic injury in premature infants occurs during a vulnerable developmental period and is associated with wider neuropathology. Acta Neuropathol. Commun..

[24] Limperopoulos C (2005). Cerebellar hemorrhage in the preterm infant: ultrasonographic findings and risk factors. Pediatrics.

[25] Steggerda SJ (2013). Small cerebellar hemorrhage in preterm infants: perinatal and postnatal factors and outcome. Cerebellum.

[26] Tam EWY (2011). Cerebellar hemorrhage on magnetic resonance imaging in preterm newborns associated with abnormal neurologic outcome. J. Pediatr..

[27] Hortensius LM (2018). Neurodevelopmental consequences of preterm isolated cerebellar hemorrhage: a systematic review. Pediatrics.

[28] Boswinkel V., Steggerda S. J., Fumagalli M., Parodi A., Ramenghi L. A., Groenendaal F., Dudink J., Benders M. N., Knol R., de Vries L. S., van Wezel-Meijler G. (2019). The CHOPIn study: a multicenter study on cerebellar hemorrhage and outcome in preterm infants. Cerebellum.

[29] Limperopoulos C (2005). Late gestation cerebellar growth is rapid and impeded by premature birth. Pediatrics.

[30] Agyemang AA (2017). Cerebellar exposure to cell-free hemoglobin following preterm intraventricular hemorrhage: causal in cerebellar damage?. Transl. Stroke Res..

[31] Jeong HJ (2016). Cerebellar development in preterm infants at term-equivalent age is impaired after low-grade intraventricular hemorrhage. J. Pediatr..

[32] Sancak S, Gursoy T, Karatekin G, Ovali F (2017). Effect of intraventricular hemorrhage on cerebellar growth in preterm neonates. Cerebellum.

[33] Imamoglu EY, Gursoy T, Ovali F, Hayran M, Karatekin G (2013). Nomograms of cerebellar vermis height and transverse cerebellar diameter in appropriate-for-gestational-age neonates. Early Hum. Dev..

[34] Davies MW, Swaminathan M, Betheras FR (2001). Measurement of the transverse cerebellar diameter in preterm neonates and its use in assessment of gestational age. Australas. Radiol..

[35] Swaminathan M, Davies MW, Davis PG, Betheras FR (1999). Transverse cerebellar diameter on cranial ultrasound scan in preterm neonates in an Australian population. J. Paediatr. Child Health.

[36] Ecury-Goossen GM (2010). The clinical presentation of preterm cerebellar haemorrhage. Eur. J. Pediatr..

[37] Martino F (2016). Prenatal MR imaging features of isolated cerebellar haemorrhagic lesions. Eur. Radiol..

[38] Glenn OA, Bianco K, Barkovich AJ, Callen PW, Parer JT (2007). Fetal cerebellar hemorrhage in parvovirus-associated non-immune hydrops fetalis. J. Matern. Fetal Neonatal Med..

[39] Ortiz JU (2004). Severe fetal cytomegalovirus infection associated with cerebellar hemorrhage. Ultrasound Obstet. Gynecol..

[40] Aziz NA (2016). Fetal cerebellar hemorrhage: three cases with postnatal follow-up. Ultrasound Obstet. Gynecol..

[41] Nomura ML, Barini R, de Andrade KC, Faro C, Marins M (2009). Prenatal diagnosis of isolated fetal cerebellar hemorrhage associated with maternal septic shock. Prenat. Diagn..

[42] Hayashi M (2015). Prenatal cerebellar hemorrhage: fetal and postnatal neuroimaging findings and postnatal outcome. Pediatr. Neurol..

[43] Limperopoulos C, Folkerth R, Barnewolt CE, Connolly S, Du Plessis AJ (2010). Posthemorrhagic cerebellar disruption mimicking Dandy–Walker malformation: fetal imaging and neuropathology findings. Semin. Pediatr. Neurol..

[44] Rooks VJ (2008). Prevalence and evolution of intracranial hemorrhage in asymptomatic term infants. Am. J. Neuroradiol..

[45] Volpe, J. J. *Neurology of the Newborn, 6th edition* (Elsevier, 2018).

[46] Sargent, M. A., Poskitt, K. J., Roland, E. H., Hill, A. & Hendson G. Cerebellar vermian atrophy after neonatal hypoxic–ischemic encephalopathy. *Am. J. Neuroradiol.* **25**, 1008–1015 (2004). PMC7975683 15205139

[47] Connolly DJA, Widjaja E, Griffiths PD (2007). Involvement of the anterior lobe of the cerebellar vermis in perinatal profound hypoxia. Am. J. Neuroradiol..

[48] Le Strange E, Saeed N, Cowan FM, Edwards AD, Rutherford MA (2004). MR imaging quantification of cerebellar growth following hypoxic–ischemic injury to the neonatal brain. Am. J. Neuroradiol..

[49] Jouvet P (1999). Reproducibility and accuracy of MR imaging of the brain after severe birth asphyxia. Am. J. Neuroradiol..

[50] Alderliesten T, Nikkels PGJ, Benders MJNL, de Vries LS, Groenendaal F (2013). Antemortem cranial MRI compared with postmortem histopathologic examination of the brain in term infants with neonatal encephalopathy following perinatal asphyxia. Arch. Dis. Child Fetal Neonatal Ed..

[51] Martinez-Biarge M (2011). Predicting motor outcome and death in term hypoxic-ischemic encephalopathy. Neurology.

[52] de Vries LS (2004). The spectrum of cranial ultrasound and magnetic resonance imaging abnormalities in congenital cytomegalovirus infection. Neuropediatrics.

[53] Steinlin M, Blaser S, Boltshauser E (1998). Cerebellar involvement in metabolic disorders: a pattern-recognition approach. Neuroradiology.

[54] Antoun H, Villeneuve N, Gelot A, Panisset S, Adamsbaum C (1999). Cerebellar atrophy: an important feature of carbohydrate deficient glycoprotein syndrome type 1. Pediatr. Radiol..

[55] Gano D (2016). Antenatal exposure to magnesium sulfate is associated with reduced cerebellar hemorrhage in preterm newborns. J. Pediatr..

[56] Parodi A (2015). Low-grade intraventricular hemorrhage: is ultrasound good enough?. J. Matern. Neonatal Med..

[57] Bouyssi-Kobar M (2016). Third trimester brain growth in preterm infants compared with in utero healthy fetuses. Pediatrics.

[58] Stoodley CJ, Limperopoulos C (2016). Structure–function relationships in the developing cerebellum: evidence from early-life cerebellar injury and neurodevelopmental disorders. Semin. Fetal Neonatal Med..

[59] Allin MPG (2016). Novel insights from quantitative imaging of the developing cerebellum. Semin. Fetal Neonatal Med..

[60] Limperopoulos C (2014). Injury to the premature cerebellum: outcome is related to remote cortical development. Cereb. Cortex.

